# Correlations between color perception and motor function impairment in children with spastic cerebral palsy

**DOI:** 10.1186/1744-9081-10-22

**Published:** 2014-06-25

**Authors:** Marcelo Fernandes Costa, Jaelsa Cunha Pereira

**Affiliations:** 1 Departamento de Psicologia Experimental, Instituto de Psicologia, Universidade de São Paulo, Av. Prof Mello Moraes, 1721, Butantã, São Paulo, SP 05508-030, Brasil; 2 Núcleo de Neurociências e Comportamento e Neurociência Aplicada, Universidade de São Paulo, São Paulo, SP, Brasil

**Keywords:** Cerebral palsy, Visual development, Cortical impairment, Color vision, Visual perception, Psychophysics

## Abstract

**Introduction:**

The aim of the present study was to evaluate color perception thresholds and relate them to the degree of motor impairment in children with spastic cerebral palsy (SCP).

**Methods:**

Binocular and monocular chromaticity discrimination thresholds were estimated for the protan, deutan, and tritan color confusion axes in 43 SCP children aged 6–15 years who were classified as tetraplegic (*n* = 12), diplegic (*n* = 16), and hemiplegic (*n* = 15) without ophthalmological complaints. Motor impairment was rated according to the Gross Motor Function Classification System (GMFCS) in five levels of severity.

**Results:**

Analysis of variance showed significantly reduced discrimination in tetraplegic children (*p* < 0.001) compared with the diplegic, hemiplegic, and control groups. We also found a positive correlation between chromaticity discrimination thresholds and GMFCS ratings in all of the groups.

**Discussion:**

Chromaticity discrimination thresholds measured psychophysically were reduced for all three color confusion axis in tetraplegic children compared with normal children. Diplegic and hemiplegic children had similar results as normal children. The finding of a correlation between quantified motor impairment and color discrimination losses in SCP patients is a new observation that might help elucidate the causes of color perception loss in these patients. Visual information is essential for the rehabilitation of CP children. Knowledge of the degree of correlation between vision and motor impairment is valuable when planning a rehabilitation program.

## Introduction

Cerebral Palsy (CP) designates a large group of motor and sensory impairments caused by a non-progressive lesion of the brain that occurs early in life [1]. These defects are permanent but may exhibit some plasticity. The most frequent and severe motor impairment in such children is spastic cerebral palsy (SCP) [2,3]. Cognitive alterations, mental retardation, epilepsy, and hearing loss are frequently associated with SCP [4]. Additionally, ophthalmological disturbances, such as oculomotor abnormalities and visual acuity loss, are often observed [5]. Higher prevalence of refractive error than in normal children has consistently been found in SCP children [6,7]. Hypoxia/anoxia insults have been associated with this higher prevalence. Cortical visual impairment is also found in children with SCP. The most often cortical damage in these children is found in retrochiasmatic visual pathways [8-10].

In children with SCP, the degree of motor impairment has been shown to be positively correlated with the loss of visual acuity, in which more severely affected children have worse visual acuity than less-affected children, measured by the Visual Acuity Card Test [11]. We confirmed these previous results by correlating visual acuity measured by sweep visual evoked potential (sweepVEP) with Gross Motor Function Classification System (GMFCS) [12] score [13-15]. Recently, visual functions related to higher-order cortical processing were also found to be impaired in SCP children. The most frequent impairments were in visually guided object recognition [16], eye-hand coordination [17], language comprehension, visuospatial abilities, and visual memory [18,19].

Although these previous studies found retrochiasmatic visual pathway impairments and visual acuity loss, no relationship was empirically observed between visual pathway impairments and the reduction of visual acuity [8]. Structural impairment has been suggested in frontal, parietal, and temporal cortical areas based on functional damage in visual tasks, including visuospatial abilities [19-21], ocular posture [22], and eye movement control during fixation [23,24]. As mentioned above, we found that grating acuity is impaired in SCP children using a sweepVEP paradigm [13-15] and behavioral methods [25].

Color perception has been previously investigated in SCP children in the works of Sakuma [26] and Kozeis et al. [27]. In both studies, color discriminations were analyzed based on simple ordering or figure-found tasks and did not found impairments in SCP children.

In the present study, we measured chromaticity discrimination in spastic CP children for the protan, deutan, and tritan color confusion axes. Our hypothesis was that chromatic vision, similar to visual acuity, is generally impaired and positively correlated with the degree of motor impairment since visual pathway damage would probably affect other functions than spatial vision. Although SCP impairment is attributable to cortical and/or subcortical damage, only post-receptoral visual pathways were expected to be affected and, in this case, a red-green, blue-yellow or general impairment.

## Methods

### Subjects

We evaluated 50 patients with SCP, ranging in age from 6 to 15 years (mean, 10.1 years; SD = 2.8 years), who were referred by the Physiotherapy Department, Medicine Faculty, University of Sao Paulo, and had received diagnoses and follow-up. This study followed the tenets of the Declaration of Helsinki. Informed consent was obtained from the subjects after the nature of the study was explained.

The diagnosis of SCP was established by clinical and neurological examination and family history. Motor performance of the patients with SCP was assessed and divided according the GMFCS, which evaluates motor function specific to SCP. The GMFCS objectively classifies children’s current gross motor function. The focus is on self-initiated movement, with a particular emphasis on sitting and walking function. Function is divided into five levels: Level I (the most independent motor function) to Level V (totally dependent on assistance) [12].

The assessment was made by physiotherapists from the Physiotherapy Department staff. Seven subjects did not meet the inclusion criteria because they had reduced attention and poor collaboration and were excluded from the analysis. A total of 43 patients with a good understanding of the study and cognitive function were selected for the study. They were classified into three groups according to motor impairment: tetraplegic (both arms and legs affected; *n* = 12), diplegic (both arms and legs affected, but more often the legs are more severely affected; *n* = 16), and hemiplegic (hemi-body affected; *n* = 15). Systematic demographic data of the 43 patients with SCP and control children are shown in Table 1.

**Table 1:** Summary of the Cerebral palsied and control children demographic data

	**Control children**	**Spastic CP children**		
		**Tetraplegic**	**Diplegic**	**Hemiplegic**
Mean Age (SD)	10.8(2.6)	9.8(3.3)	10.8(2.7)	9.3(3.1)
Gender				
Male	n = 26	2 = 9	n = 10	n = 8
Female	n = 27	n = 3	n = 6	n = 7
Color Thresholds*				
Binocular				
Protan axis	46.8(13.8)	310.0(384.1)	90.2(42.8)	69.2(40.9)
Deutan axis	42.9(9.4)	442.6(491.3)	80.4(41.7)	67.6(35.8)
Tritan axis	67.5(18.4)	258.0(147.7)	93.7(51.1)	99.2(48.8)
Monocular OD				
Protan axis	46.2(14.2)	270.6(277.7)	98.2(59.6)	77.2(62.9)
Deutan axis	48.1(15.6)	262.4(291.5)	89.9(37.8)	75.1(31.7)
Tritan axis	65.8(16.1)	250.4(247.7)	111.6(72.1)	103.0(57.9)
Monocular OS				
Protan axis	47.6(16.0)	299.6(342.9)	85.6(41.7)	81.8(60.9)
Deutan axis	48.6(14.7)	396.2(423.3)	79.6(35.1)	68.5(30.3)
Tritan axis	64.8(17.2)	307.2(281.6)	108.8(61.4)	100.6(44.6)

*distance in CIE 1976 u’ v’ units.

An ophthalmological examination was performed for all of the subjects to eliminate confounding pathologies, such as cataracts, retinopathy, and neuropathy. Fundoscopy was performed using indirect ophthalmoscopy. Grating acuity was measured at 57 cm using Teller Acuity Cards (Vistatech, Tulsa, USA). All of the patients had normal eye fundus and 20/50 or better best-corrected visual acuity. The control group consisted of 53 healthy subjects, ranging in age from 6 to 16 years (mean, 10.5 years; SD = 2.8 years). The inclusion criteria for this group were normal eye fundus and 20/20 or better best-corrected visual acuity.

### Equipment and procedures

The evaluation of color discrimination was performed using a color vision test for children (adapted by Goulart et al. [28]), based on the commercial version of the Cambridge Colour Test (CCT 2.0, Cambridge Research Instruments, Cambridge, UK) [29] installed on a personal computer (Dell Dimension XTC-600) with a VSG 2/5 graphic board (Cambridge Research Instruments, Cambridge, UK). The stimuli were generated on a high-resolution color monitor (Sony FD Trinitron model GDM-F500T9). Testing was conducted in a dark room with the subjects positioned 2.5 m away from the monitor.

The stimulus provided by the CCT for Kids was similar to those used in the pseudoisochromatic plate tests, such as the Ishihara test (Kanehara & Co.) and AO H-R-R (Richmond Products). The target consisted of a square that differed in chromaticity from the single neutral background (coordinates of the International Commission on Illumination [CIE] u’v’ 1976 color space: 0.1977, 0.4689). The square size corresponded to 1.25 degrees of visual angle at a test distance of 2.5 m. Both the target and background were composed of small patches of varying sizes (0.5-2 cm diameter) and six luminance levels (8, 10, 12, 14, 16, and 18 candela [cd]/m^2^) randomly distributed on the display. This design uses spatial and luminance noise to avoid the influence of cues derived from luminance differences or from target contours in the intended hue discrimination.

**Figure 1:**
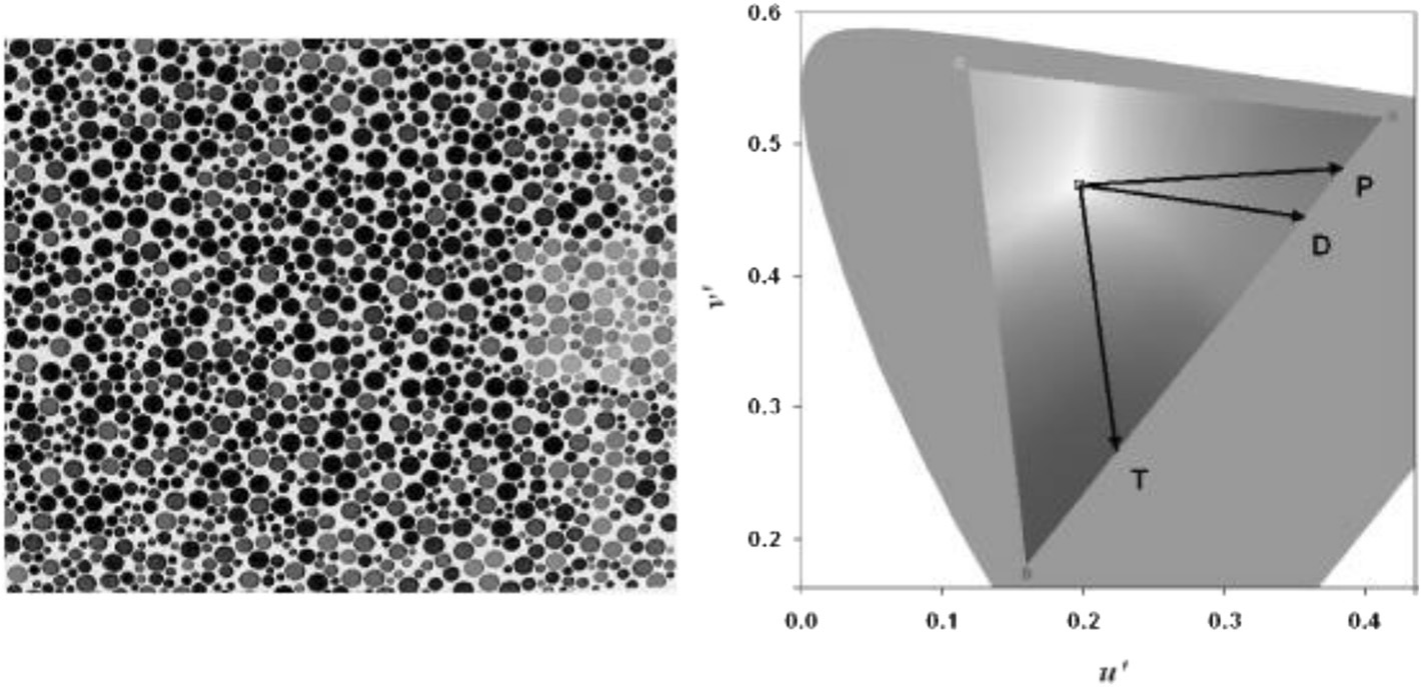
**(Right) The 1976 CIE u’v’ color diagram used by the Cambridge Colour Test.** (Left) An image of the stimulus provided by the CCT showing the isoluminant noise and letter C (left). The gray area indicates all of the colors seen by the human visual system, and the white triangle indicates all of the colors used by the monitor for this luminance level. The P, D, and T lines correspond to the protan, deutan, and tritan confusion axes tested in the Trivector protocol.

The target was randomly presented in one of two positions: right and left (2-Alternative Forced Choice strategy). The patient’s task was to indicate the side on which the colored squared was, and the examiner pressed one of two buttons of the response box (CT3; Cambridge Research Instruments, Cambridge, UK) that corresponded to the position of the square. Verbal, eye, or head movements were used as a response, depending on the children’s abilities. The subjects had up to 15 s to emit the response. Because some of the patients could have strabismus (i.e., a frequent comorbidity in CP that can reduce visual acuity in one eye), the color measurements were performed under three conditions: binocularly and monocularly for the right and left eyes.A psychophysical staircase procedure was used for threshold determination along the protan, deutan, and tritan confusion axes (Figure 1). The color confusion axes are lines in the color space in which color-blind subjects have worse discrimination by confounding the saturated color with a level of gray. In this procedure, the three corresponding staircases are conducted simultaneously in an interleaved way, changing randomly from one to the other. Each staircase began with a saturated chromaticity that was changed along the vector that connected it to the background chromaticity. Periodically, a control target at maximum saturation was presented as a catch trial.

The change depended on the patient’s response. The target chromaticity approached the background chromaticity every time there was a correct response and moved away from it every time there was an incorrect response or no response. The chromaticity excursion along the vectors ranged from 0.1100 to 0.0020 units of CIE 1976 u’v’. After 11 staircase reversals, the program automatically calculated the threshold for that vector as the average of the chromaticities that corresponded to the last six reversals. The step size used in the staircase followed a dynamic rule (for more details on the CCT methodology, see Regan et al. [29]; for the CCT norms, see Paramei [28-30]).

### Response reliability

The CCT software incorporates a reliability-testing procedure with catch trials that present a saturated color at the maximum of the cathode ray tube gamut. These catch trials are presented at different times in the test session and constitute approximately 10% of the stimuli. The color used in the catch trial was a highly saturated light blue that did not correspond to any of the color confusion axes (CIE 1976 u’v’ coordinates: 0.119, 0.391; vector length: 1,100 u’v’ units). That saturated color is discriminated even by patients with severe congenital and acquired color vision impairment. This procedure tests the ability of the subject to respond correctly to the target, which depends on understanding the instructions and attention directed to the task during the test session. We defined the percentage of correct responses in these catch trials as a measure of reliability. Reliability was 100% for both control subjects and patients with CP (i.e., no mistakes were made in the catch trials). This means that the subjects were performing the required task correctly during the entire duration of the test session.

### Statistical analysis

The statistical analysis was performed using Statistica 11 software (StatSoft, Tulsa, OK, USA). A complete descriptive analysis was performed. Adherence to a normal distribution of the data was checked using the Kolmogorov-Smirnov test. Statistical differences among groups were verified using repeated-measures analysis of variance (ANOVA), with SCP type as a factor. We used the Fisher *post hoc* test to determine significant differences between group means in the ANOVA. The Pearson correlation test was used to verify the relationships between the color vision results and the other variables (i.e., age and GMFCS score).

## Results

We were able to measure the chromaticity thresholds psychophysically in all of the children with SCP. Statistical differences were found between the tetraplegic children and other SCP and control groups for the three color confusion axes and both of the visual conditions (i.e., monocular and binocular). The statistical summary is presented in Table 2.

**Table 2:** Statistical differences between the cerebral palsied and control children

	**Spastic CP Children**		
	**Tetraplegic**	**Diplegic**	**Hemiplegic**
Color Thresholds			
Binocular			
Protan axis	F = 14.93 p = .003	ns*	ns
Deutan axis	F = 21.87 p < .001	ns	ns
Tritan axis	F = 26.57 p < .001	ns	ns
Monocular OD			
Protan axis	F = 16.75 p = .004	ns	ns
Deutan axis	F = 16.87 p = .002	ns	ns
Tritan axis	F = 12.67 p < .002	ns	ns
Monocular OS			
Protan axis	F = 15.73 p = .002	ns	ns
Deutan axis	F = 20.07 p < .001	ns	ns
Tritan axis	F = 19.29 p < .001	ns	ns

*non significant from the control group.

**Figure 2:**
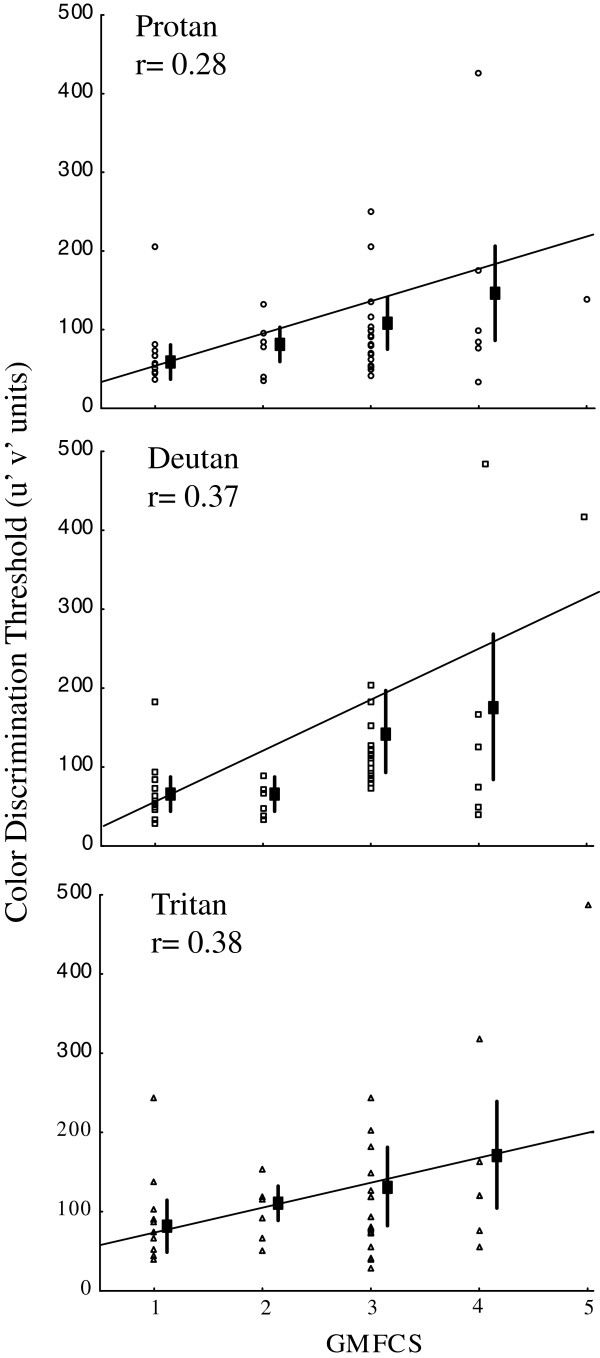
**Correlation between the levels of motor impairment classified by the Gross Motor Function Classification System (GMFCS) and color discrimination thresholds for the binocular condition.** A positive correlation was found in all of the spastic cerebral palsy subgroups.

**Figure 3:**
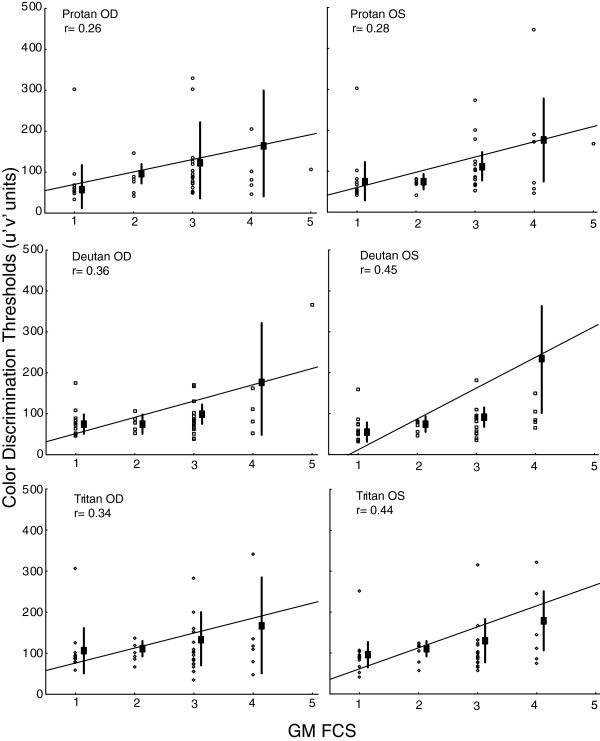
**Correlation between the levels of motor impairment classified by the Gross Motor Function Classification System (GMFCS) and color discrimination thresholds for the monocular condition.** A positive correlation was found in all of the spastic cerebral palsy subgroups.

**Figure 4:**
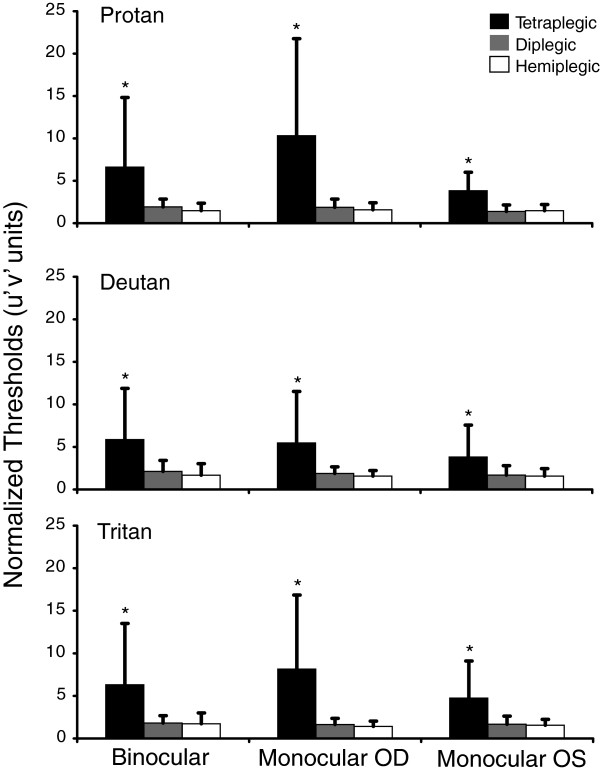
**The magnitude of color discrimination loss in the three motor impairment groups for the binocular and monocular conditions.** Tetraplegic children had higher color vision impairment, with five-times less discrimination than controls. Diplegic and hemiplegic children had similar losses, with an average of two-times worse discrimination.

A small negative correlation was found between age and chromaticity thresholds in the control group, suggesting an increase in color discrimination performance with age. The opposite result was found for the SCP children, in which a positive correlation was found for all of the visual conditions. The hemiplegic group had almost no correlation. These data are summarized in Table 3.Positive correlations were also found between chromaticity thresholds and GMFCS scores within groups, indicating that worse motor impairment was associated with worse color discrimination (see Figure 2 for binocular data and Figure 3 for monocular data).The magnitude of color discrimination loss was obtained by normalizing the data from SCP children. Tetraplegic children had higher color discrimination loss, with an average of five-times worse discrimination than control children. Diplegic and hemiplegic children had similar losses, with an average of two-times worse discrimination (Figure 4).

**Table 3:** Color discrimination thresholds and age correlation coefficients

	**Binbocular**	**Monocular OD**	**Monocular OS**
Correlation Coefficient*			
Controls			
Protan axis	**r = -0.11 (0.041)**	**r = -0.16 (0.023)**	r = -0.06 (0.066)
Deutan axis	**r = -.023 (0.008)**	**r = -0.11 (0.040)**	**r = -0.17 (0.021)**
Tritan axis	**r = -0.14 (0.031)**	r = -0.04 (0.074)	**r = -0.18 (0.019)**
Tetraplegic			
Protan axis	**r = 0.70 (0.041)**	**r = 0.69 (0.033)**	**r = 0.90 (0.033)**
Deutan axis	**r = 0.30 (0.038)**	**r = 0.10 (0.045)**	**r = 0.10 (0.056)**
Tritan axis	**r = 0.00 (0.036)**	r = 0.06 (0.079)	r = 0.01 (0.109)
Diplegic			
Protan axis	**r = 0.48 (0.047)**	**r = 0.30 (0.053)**	**r = 0.34 (0.039)**
Deutan axis	**r = 0.42 (0.049)**	**r = 0.49 (0.042)**	**r = 0.26 (0.045)**
Tritan axis	**r = 0.43 (0.038)**	**r = 0.36 (0.043)**	**r = 0.35 (0.041)**
Hemiplegic			
Protan axis	r = 0.36 (0.066)	r = 0.25 (0.079)	**r = 0.04 (0.049)**
Deutan axis	r = 0.33 (0.052)	r = -0.07 (0.067)	r = 0.05 (0.064)
Tritan axis	r = 0.11 (0.061)	r = 0.21 (0.059)	r = 0.10 (0.061)

*Correlation coefficient (p-value).

No differences were found between the visual conditions (monocular and binocular) in control and SCP children. Also no differences were found within SCP group considering etiology and prematurity.

## Discussion

Color vision is impaired in SCP children. We evaluated chromaticity discrimination along the protan, deutan, and tritan color confusion axes. In all of these axes, discrimination thresholds were reduced compared with control children. The tetraplegic group was the most affected, exhibiting higher thresholds compared with the diplegic and hemiplegic groups. To our knowledge, no studies have evaluated color vision in SCP children. Our study has important implications because cerebral palsy has been studied for a long time, with much data generated about the visual changes associated with ophthalmologic strabismus and lens accommodation paresis findings [6,8,9,11,31-37].

Visual acuity is classically reduced in SCP children. Recent studies have shown that it is positively correlated with motor function measured by the GMFCS [13-15,25,32,34,37]. Similar to previous visual acuity studies, we found a positive correlation between chromaticity discrimination thresholds and GMFCS scores within each motor group condition. We argue that the visual acuity reduction in our sample did not interfere with color vision because the worst visual acuity of our sample (20/50) means a discrimination threshold of 2.5 cycles per degree of visual angle. The stimulus had 1.25 degrees (or 46 minutes of arc), which was sufficiently wide to be seen by our children.

The color vision impairment observed in the present study was also related to the motor condition. Tetraplegic children had worse function compared with diplegic and hemiplegic children. Hemiplegic children had similar color vision as control children. Better visual and cognitive functions in hemiplegic children have been described in previous studies [9,11,15,32,35]. This finding corroborates studies of motor function that reported less neurological impairment compared with diplegic and tetraplegic children [2,4,18].

The developmental characteristic of chromaticity discrimination was assessed in the present study. We found that chromaticity discrimination is under development because the children in the control group exhibited a negative correlation between age and chromaticity thresholds obtained in each color confusion axis. This means that older children had lower thresholds. This is consistent with the findings of other studies that suggested that chromaticity discrimination is fully developed at the end of adolescence [30,31,38,39].

One way to evaluate the development of chromaticity discrimination in SCP is to assess whether the threshold values decrease in older children. Considering the age of the children evaluated in the present study, our hypothesis is that, even with overall deficits in chromatic discrimination, the development of that function was occurring. However, we found a completely opposite result. As age increased, chromaticity thresholds increased, suggesting a progressive reduction of color vision discrimination. Thus, in children with SCP, color vision is both reduced compared with normal children and developmentally impaired. One possible interpretation could be related to sensorial impairment that affects the increase in perceptual complexity. Studies have demonstrated that multisensorial and associative cerebral areas are the most sensitive to external stimulation [40,41]. Some associative areas, such as the dorsolateral prefrontal cortex, have been considered to subserve critical cognitive abilities during early infancy, and improvements in these abilities are evident over roughly the next 10 years. These authors found a reduction of visual contrast sensitivity function in children with diseases that affect the dorsolateral prefrontal cortex. Thus, damage to sensory and perceptual systems early in life may interfere with chromatic discrimination function in a complex way and interfere with the course of their development. We also demonstrated that visual acuity is also impaired in SCP children [13-15,25]. The same cortical mechanism may be involved in both functions.

We evaluated the reliability of the test by computing the correct responses to the catch stimulus. If effects on cognitive function were affecting our measurement of chromaticity discrimination, then the reliability of the responses in the catch trial could be important. Because we had 100% correct responses in the catch trials for all of the subjects under all of the conditions, we can consider that the reduction of chromaticity discrimination reflected impairment in sensorial pathways.

Etiology and prematurity were not variables that affected chromaticity discrimination. Perinatal anoxia was the most frequent etiology, consistent with other studies [4,42,43]. However, no differences were found between the children with anoxia and those with another etiology, thus corroborating previous studies in which etiology was unrelated to visual impairments in children with SCP [8-10,43-45].

Similar to the control group, the SCP children showed no difference in chromaticity discrimination thresholds in the monocular and binocular viewing conditions. These absences of a binocular summation had been reported to chromaticity discrimination function in normal subjects using the commercial version of the CCT [46].

There are some limitations that we consider critical for the interpretation of our findings. The need for a minimum level of cooperation from the patient may be underestimating the defect in color vision associated with the SCP. Psychophysical measures by involving behavioral, verbal or motor responses could certainly impair threshold measurements in different populations such as that we studied. The impaired motor behavior in the SCP children certainly adds a level of uncertainty that could not be precisely estimated. Although all controls performed to ensure the validity of the measures as measure reliability for the catch trials, the uncertainty in the threshold level may have been influenced by the lack of a clear and accurate motor behavior.

In conclusion, color perception based on chromaticity discrimination is generally reduced in SCP children. Tetraplegic children had worse color discrimination than the other two SCP groups (diplegic and hemiplegic). The impairment in chromaticity discrimination was proportional to GMFCS score for all SCP motor impairment subgroups.

## Competing interests

The authors declare that they have no competing interests.

## Authors’ contribution

All authors (MFC and JCP) participated to the design of the study. MFC supervised and the JCP performed the experiments. MFC performed the statistical analyses and drafted the manuscript. Both authors read, amended and approved the final manuscript.
